# Substance Use Among Gay Men Living in Mexico: Perceptions of Well-Being and Implications for Health

**DOI:** 10.1007/s13178-025-01120-9

**Published:** 2025-03-29

**Authors:** Juan Carlos Mendoza-Pérez, Héctor Alexis López-Barrientos, Michael P. Dentato

**Affiliations:** 1 Faculty of Medicine, Department of Public Health, National Autonomous University of Mexico, Av. Universidad 3000, 04510 Mexico City, Mexico; 2 Faculty of Political and Social Sciences, National Autonomous University of Mexico, Mexico City, Mexico; 3 School of Social Work, Loyola University Chicago, Chicago, USA

**Keywords:** Substance use, Gay men, Harm reduction, Well-being, Sexualized drug use

## Abstract

**Introduction:**

Studies on substance use among gay men are often analyzed from the framework of risk and harm, disregarding the user’s perspective about various reasons for use, which for some may not be perceived as potentially harmful. The current study examines substance use among gay men and perceived benefits and adverse effects on their well-being from three unique geographical areas across Mexico.

**Methods:**

A mixed methods survey and three online focus groups were conducted in May 2022. Nineteen gay men (average age 32 years) from northern, central, and southern Mexico participated in the study. Data was interpreted utilizing a content analysis.

**Results:**

Poppers, crystal meth, and marijuana were the most recently consumed substances, with frequent use reported during sex. First-contact drugs and recreational drugs showed greater perceived social and mental health benefits. In contrast, drugs used during sex had greater perceived adverse effects on overall well-being, primarily with the use of crystal meth.

**Conclusions:**

There is an apparent ambivalence with using drugs based on the experiences of gay men participating in this study who live in Mexico. Some participants reported an improvement in mental health and emotional well-being, sexual experiences, and increased socialization. Other participants reported harmful effects upon their health, mental health, and social well-being based on decisions to use substances. Implementing a gay-affirming harm reduction model may improve overall health outcomes for this minority population, as well as their mental health, overall well-being, and care needs.

**Supplementary Information:**

The online version contains supplementary material available at 10.1007/s13178-025-01120-9.

## Introduction

Substance use among gay men is an ongoing issue that has relevance for public health, education, and prevention strategies due to the many risks faced by this minority population. Such risks may include sexually transmitted infections (STIs) such as HIV or be a result of oppression; trauma; and violence due to the sociopolitical climate and other forms of societal, institutional, or community-based discrimination (Schroeder et al., 2022). Recent international literature focuses on the harmful effects of substance use related to several sexual risk factors (e.g., HIV, STIs, unprotected anal sex) and adverse outcomes associated with psycho-emotional health due to sexualized drug use, including depression, anxiety, substance use dependence, and suicide ideation (Hibbert et al., 2021; Íncera-Fernández et al., 2021).

Several surveys conducted in 2015 assessed substance use among gay men in Mexico in terms of prevalence, frequency, and types of substances. Results demonstrated that between 74 and 88% of gay male participants actively consumed alcohol, and just under 46% used tobacco (Baruch et al., 2015; Mendoza-Pérez et al., 2015). In contrast, nearly half of gay men in both studies had consumed a substance (apart from alcohol or tobacco) at some time in their lives, with most frequently used substances including (in descending order) marijuana, inhaled nitrites (poppers), cocaine, and methylenedioxymethamphetamine (MDMA, ecstasy) (Baruch et al., 2015; Mendoza-Pérez et al., 2015).

Another study conducted during 2017–2018 among 1850 men who have sex with men (MSM) attending HIV/STI clinics in Mexico City reported that alcohol (76.2%) and tobacco (29.2%) were the most consumed substances in the past month with notable use of marijuana (29.4%), sexual drugs (24.2%), stimulants (13.7%), and depressants (6.3%) (Rodríguez-Bolaños et al., 2022). This is consistent with what was reported about other substance use during the COVID-19 global health pandemic, in which marijuana, poppers, and crystal meth were the three substances most consumed by gay men in Mexico, followed by cocaine, tranquilizers, and MDMA also known as “molly” (Mendoza-Pérez, 2021).

Similar to other studies in this area, most Mexican researchers frame substance use among gay men within a risk paradigm, which includes HIV transmission and condomless anal sex, but also based on certain sociodemographic factors or sociosexual practices while using substances (e.g., serosorting, which is selecting partners with the same HIV status for condomless anal sex) that may have implications for sexual health (Algarin et al., 2023; Caballero-Hoyos et al., 2022; Mendoza-Pérez et al., 2022; Rodríguez-Bolaños et al., 2022). For example, in binational studies of Mexican/US border cities such as Ciudad Juárez and San Diego-Tijuana, MSM who inject drugs (Deiss et al., 2008), use crystal meth (Loza et al., 2020), or use poppers (Pepper et al., 2020) were found to engage in various risk behaviors (e.g., needle sharing, transactional sex for money or drugs, unprotected anal sex) in addition to being at increased risk of STI transmission (e.g., HIV, syphilis, gonorrhea).

## Additional Perspectives for Addressing Substance Use Among Gay Men

The World Health Organization (WHO) maintains that health, “…is a state of complete physical, mental and social well-being and not merely the absence of disease or infirmity…”(World Health Organization, 2020, p. 1). As Halbreich (2022) explains, this optimal state of well-being includes physical and emotional health, daily functioning, and interactions with institutions and the broader community, providing an integrative perspective for biopsychosocial health. This offers a view of how to navigate complex physical and sexual risk factors, but it does not delve into relevant key variables (e.g., age, race, sexual orientation) that may help to explain the impact of substance use upon well-being.

In that regard, the model of evaluating substance use environments utilized by gay men in Mexico proposed by Mendoza-Pérez et al. (2023) delves into the biopsychosocial implications of substance use by assessing personal experiences (i.e., motivations, types of substances, forms, and contexts of use) based on changes in consumption patterns and perceived impact upon well-being. The model is not presented as a linear sequence or mutually exclusive environments (i.e., as gay men have different starting points with substances or move through environments differently) but rather by assessing the uniquely lived experiences of these men at various moments in their lives.

This is relevant in that substance use among gay men does not depend solely upon external factors but also on how associated health-disease processes may be a product of genetics, social relationships, and/or cultural processes (Martínez Hernáez, 2007). As Apud and Romaní (2016) explain from the sociocultural model, substance use is interpreted as a field of intersubjective relations between objects, subjects, and cultural contexts where consumption acquires particular meaning for constructing identity and organizing daily life. In other words, this paradigm challenges the purely biological notion of addiction, seeking to understand the phenomenon from the perspective of users about the surrounding stigmatized, pathological, and illegal contexts, helping to establish better strategies for healthcare education and prevention methods (Alvarez Licona, 2010; Romaní, 2007), which is all closely linked to harm reduction.

A harm reduction approach is among the key prevention strategies to reduce harm (rather than stop substance use per se) as it considers what an individual finds harmful or beneficial rather than emphasizing risk behavior in moral terms of right or wrong (Marlatt, 1996; Ritter & Cameron, 2006). Research conducted at an LGBT harm reduction treatment center in Sydney, Australia, showed positive outcomes and improved psychosocial well-being for gay and bisexual crystal meth users. Participants reported lower use and dependence after free, short-term, individual outpatient counseling using a culturally sensitive LGBTQ peer-led model recognizing the historical-social context of substance use and inequalities faced in healthcare institutions (Lea et al., 2017).

Birch et al. (2022) examined a US sample of private sex party hosts as “gatekeepers” who promoted harm reduction strategies before the events for gay and bi men which included screening participants, disseminating rules about sexual behaviors and substance use, reinforcing rules, providing condoms and lube, specifying which substances were allowed, and turning away uninvited guests, suggesting that these men may be the focus of group-based sexuality education and intervention programs. Relatedly, Zucker et al. (2022) evaluated a four-hour course (“Party Keepers”) aimed at Israeli gay men who frequent LGBT parties to identify, prevent, and treat medical emergencies associated with excessive substance use. Researchers found a reduction in the use of alcohol, cocaine, and risky sexual practices by study participants and less likelihood of emergencies at such events, in addition to an increase in the use of pre-exposure prophylaxis (PrEP) by course attendees. Other intervention strategies for gay men who use substances, such as cognitive behavioral therapy (CBT), have been shown to reduce the likelihood of unprotected anal sex (Melendez-Torres & Bonell, 2014). However, this systematic literature review suggests that CBT is not more effective than other intervention approaches (Melendez-Torres & Bonell, 2014), like community-based harm reduction strategies that include in-person or online counseling led by peers or clinicians; distribution of safe sex packets, STI, and substance use information; and clinical screenings (Hugo et al., 2018; Stardust et al., 2018).

From this perspective, some studies analyze not only substance use risk among gay men but also the motivations and non-harmful effects, whether related to use among peers or living in hostile environments, and so on. Thus, substance use among gay men may also be seen as a survival mechanism for navigating stressful events (e.g., HIV diagnosis, discrimination, homophobia, death of a family member) and/or to improve social connections with other gay men to find acceptance, inclusion, emotional closeness, improved self-esteem, or increased sexual pleasure and satisfaction (Lafortune et al., 2021; Nimbi et al., 2021). As Romaní (2007) explains, the abstinence paradigm is not only insufficient but may also be detrimental to biopsychosocial well-being (e.g., increase in HIV transmission or other health and mental health problems arising from secretive substance use); therefore, as an impactful alternative, the harm reduction model can offer not only effective biomedical strategies yet social and cultural sensitivity toward diverse populations.

It may be helpful to examine perceptions of well-being among substance-using Mexican gay men, along with the benefits and adverse effects of substance use, as it will critically contribute to a greater understanding of behaviors beyond a framework of risk. Such an examination of gay men in Mexico may result in improving best practices in culturally affirming health and mental health care based on respect for human rights and autonomy. Therefore, the objective of this study is to examine substance use among gay men from three unique geographical areas in Mexico and the perceived benefits and adverse effects upon their well-being.

## Methods

A mixed-method study utilizing an online survey and focus groups examined substance use among gay men living in Mexico to understand their lived experiences and perceptions of well-being (Denzin & Lincoln, 2012). From the perspective of social constructionism, this study sought to understand the meanings associated with substance use as a result of social interactions and communication between gay men living in Mexico, which may be impacted by several factors including peer and societal beliefs, personal values, and knowledge about substance use (Berger & Luckmann, 2003; Maines, 2000).

Three focus groups using semi-structured interviews were held online in May 2022 via Zoom from three geographical regions of Mexico, including the northern region (Baja California, Baja California Sur, Chihuahua, Nuevo León, and Tamaulipas), the central region (Mexico City, Estado de Mexico, Puebla), and the southern region (Veracruz, Yucatán, and Quintana Roo). In addition to the focus groups, an online survey was conducted via *Qualtrics* in which sociodemographic data, types of substances consumed, and frequencies and forms of consumption were investigated.

### Participant Criteria

Participant inclusion criteria included identification as a cisgender gay man, 18 + , having used a substance (apart from tobacco and alcohol) in the past month, and residing in one of the three geographic regions mentioned above. Participants were recruited via social media (Instagram, Facebook, and X) and with the support of Inspira Cambio A.C., a community-based service provider working with sexual and gender-diverse populations in Mexico that use substances.

Nineteen gay male participants were identified to participate in the online focus groups, with the majority residing in Mexico City. The average age of study participants was 32 years. The majority had bachelor’s or postgraduate degrees (95%), and their main occupations included owning a business or simultaneously working and attending school (58%). The level of education for the head of the family was mainly at the university level (58%). Most participants received government health services (79%), 68% were living with HIV, and 16% were taking pre-exposure prophylaxis (PrEP) at the time of the study (Table 1).

**Table 1:** Sociodemographic characteristics of the sample (*N* = 19)

Variable	%
**Geographic area**	
North	26.3
Center	57.9
South	15.8
**Education: participants**	
University incomplete	5
University	74
Postgraduate	21
**Education: head of family**	
High school	21.1
University	57.9
Postgraduate	21.1
**Occupation**	
Study	10.5
Study and work	26.3
An employee of any level	21.0
Own business	31.6
Not presently working	10.5
**Type of health service accessed**	
Private service	15.8
Government service	79.0
No health services	5.3
**Men with HIV**	68.4
**Pre-exposure prophylaxis (PrEP) use in men without HIV (*N* = 6)**	50

### Analysis and Coding

Each of the three online focus groups lasted between 90 and 120 min. Recordings were transcribed, and analysis and coding were conducted using NVivo, adhering to the principles of directed content analysis (Hsieh & Shannon, 2005). Researchers used deductive analysis to focus on the meanings associated with participant language and responses. This process sought to validate and refine gaps in a theoretical paradigm or conceptual framework by identifying unique themes and categories (Hsieh & Shannon, 2005).

Contextual information was analyzed by creating a codebook based on two main categories, as perceived by the gay male participants, which included (1) the adverse effects of substance use and (2) the benefits of substance use. To explore responses to these categories, participants were asked about their perceptions of the positive aspects (e.g., *What have been the benefits to your well-being from using drugs?*) and negative aspects *(*e.g., *What have been the adverse effects to your well-being from using drugs?)* of substance use.

### Categorical Analysis

While engaging in the qualitative portion of this study, the categories of well-being, adverse effects, and perceived benefits of substance use were based upon the participant’s perceptions of their health-disease processes and not via a clinical, biomedical, or psychological assessment or diagnosis.

#### Well-being

As defined by the World Health Organization (World Health Organization, 2020) and Halbreich (2022), well-being is a state in which people and social groups may describe their biopsychosocial needs while living a life filled with satisfaction and opportunities in the face of oppression and tensions stemming from society (e.g., homophobia, heteronormativity).

#### Adverse Effects

The category of adverse effects refers to the perceived harmful, damaging, or bothersome impact of substance use upon one’s biological, psychological, and social well-being on both a personal level and at the level of social groups and networks in which gay men belong.

#### Perceived Benefits

The category of perceived benefits refers to the perceived positive or beneficial effects of substance use upon the biological, psychological, social, and economic well-being on both a personal level and at the level of various social groups and networks in which gay men belong.

#### Substance Use Environments

This study examined four substance use environments. The first is the *initiation drug environment (IE)*, which is characterized by first-time, experimental, and sporadic use of alcohol, tobacco, and/or marijuana, with unknown or few effects upon well-being (as it captures first-time use). The second is the *recreational drug environment (RE)*, which is characterized by primarily social and intermittent use of LSD (lysergic acid diethylamide), hallucinogenic mushrooms, dimethyltryptamine (DMT), ecstasy, marijuana, and/or alcohol, with some effects on well-being. The third is the *sexual drug environment (SE)*, which is characterized by the social, sexual, and/or sporadic use of inhaled nitrites (poppers), marijuana, cocaine, ketamine, sildenafil, crack, alcohol, and ethyl chloride, with more frequent and significant effects upon well-being. The final category is the *greater sexual pleasure drug environment (GE)*, which is characterized by the high-frequency use of crystal meth through smoking, inhalation, rectal or other non-conventional administration, or injection, leading to significant adverse effects on well-being (Mendoza-Pérez et al., 2023).

## Results

The results section is organized into three parts. In the first section, the types of substances and prevalence of use are described for lifetime use and use in the past month. In the second section, a list of perceived adverse effects and benefits of substance use is presented concerning the four substance use environments described above by Mendoza-Pérez et al. (2023). Finally, the third section examines the perceived adverse effects or benefits via qualitative participant responses. Please note that the analysis of perceived positive and negative impacts of substance use spans the physical, mental, and social domains of participant health and well-being.

### Types and Prevalence of Substance Use

The main substances used by gay male study participants at some point in life were crystal meth, poppers, marijuana, and ecstasy. Regarding current usage (past 30 days), poppers, crystal meth, and marijuana were most frequently used, with the latter two having the highest proportion of daily use. Regarding the use of injected substances, just under half (47%) of gay male respondents reported use in the past year. Participants reporting substance use during sex had higher frequencies in the categories of *very frequent* or *always* (64%) (Table 2).

**Table 2:** Description of frequency and use of substances

			**Substance use in the past 30 days (%)**			
	**Substance use at some time in life (%) *N* = 19**	***N***	**No substance use**	**Substance use from time to time**	**Substance use two to three times a week**	**Daily substance use**
**Types of drugs**						
Marijuana, weed, *“churro”*	84	16	37.5	18.8	25	18.7
Crystal meth	100	19	10.5	42.1	21.1	26.3
Ecstasy “*tachas*”	84	16	68.8	25	6.2	
Thinner, resistol, solvent	5	1	100			
Crack	47	9	88.9	11.1		
Cocaine, *“perico”*	68	13	46.1	46.2	7.7	
Hallucinogenic mushrooms, peyote, mescaline	58	11	72.7	18.2	9.1	
Poppers	100	19	21.1	52.6	26.3	
Ethyl chloride	58	11	63.6	27.3	9.1	
Ketamine, gamma-hydroxybutyrate (GHB)	47	9	78	22		
Amphetamines	16	3	100			
Tranquilizers without a prescription	26	5	60	40		
Sedatives or barbiturates without a medical prescription	11	2	100			
**Substance use during sexual relations**						
Seldom	10.5					
Sometimes	26.3					
Very often or always	63.2					
	**Use in the past year (%) *N* = 19**					
Substance use injected	47.4					

### Overall Adverse Effects and Perceived Benefits of Substance Use in Gay Men

This section presents results via the frameworks of three environments where substance use occurs. The first describes the effects and benefits of the *initiation drug environment* (IE), *recreational drug environment* (RE), and *sexual drug environment* (SE). In these three environments, a more significant presence of experiences linked to perceived benefits was reported, especially with the use of marijuana, recreational drugs, and inhaled nitrites (poppers), compared to the perception of adverse effects that were mainly concentrated in the substances used for sexual practices (Table 3).

**Table 3:** Adverse effects and perceived benefits in the initiation drugs, recreational drugs, and drugs for sexual purposes environments

	**Domain**	**Substance used**		**Perceived effect on health and body**	**IE**^a^	**RE**^b^	**SE**^c^
Adverse effects	Physical	Poppers		Headaches, tachycardia			x
		Marijuana		Cough, dry mouth and eyes	x		x
		Cocaine “*perico*”		Nose and throat irritation			x
		Ethyl chloride		Fainting			x
		Gamma-hydroxybutyrate (GHB)/ Gamma-butyrolactone (GBL)		Seizures			x
	Mental	Ethyl chloride		Hallucinations			x
	Social	Ethyl chloride		Deterioration of couple relationships			x
		Alcohol		Excessive consumption makes it impossible to have sexual relations	x		x
Perceived benefits	Physical	Marijuana		New ways to explore the body and sexuality, increase appetite, improve physical performance	x		x
		Poppers		Euphoria and increased sexual desire, greater anal dilation			x
	Mental	Marijuana		Management of anxiety, stress, and panic attacks; feeling of tranquility, relaxation, happiness; euphoria; increased sensory perception; creativity	x		x
		Tobacco		Anxiety management	x		
	Social	Recreational substances	Marijuana	Non-sexual socialization, improve sexual experience, optimize work and housework	x		x
			Ecstasy “*tachas*”	Non-sexual socializing, parties		x	
			Alcohol	Non-sexual socializing, parties	x	x	
			Tobacco	Social status, non-sexual socialization	x		
			Hallucinogenic mushrooms	Non-sexual socializing, parties		X	
			LSD	Non-sexual socializing, parties		x	

^a^Initiation drug environment (IE)

^b^Recreational drug environment (RE)

^c^Sexual drug environment (SE)

The second framework (Table 4) condenses the perceived benefits and adverse effects that gay male participants experienced using crystal meth. Although the use of this substance is framed within a sexual context, the main reason why it is considered a unique substance use environment (aside from the three above) is that more significant adverse effects were reported on physical, mental, and social well-being that surpassed other substances and environments. For participants, the intensity and frequency of crystal meth use was elevated due to erotic and sexual motivation, especially to achieve greater pleasure.

**Table 4:** Adverse effects and perceived benefits of crystal meth use

	**Type**	**Perceived effect on health and body**
**Negative effects**	Physical	Weight loss and lack of appetite
		Insomnia
		Tachycardia
		Nausea and vomiting
		Changes in urine (dark, foul-smelling)
		Excessive sweating
		Erectile dysfunction
		Tremors
		Muscle pain
		Constipation
		Bruxism
		Due to smoking: burns in the mouth
		Due to inhaled use: irritation of the nasal passages
		Due to injected use: injuries to the arm and vein
		Hyperthermia
		Hepatic injury
		Polyuse (crystal, sildenafil, and poppers): seizures
	Mental	Anxiety and panic attacks
		Mental gaps
		Auditory, visual, and sensory hallucinations
		Psychotic breaks
		Suicide ideation
		Fear
		Distress
	Social	Deterioration of sexual relationships and breaking up, physical violence, loss of trust, insecurity in relating emotionally and sexually
		Deterioration of family relationships: physical violence, loss of trust, theft, family concern for the person’s well-being
		Deterioration of the social environment: loss of employment, abandonment of studies
		Violence in spaces of sexual encounters: theft, verbal and physical attacks, psychological violence, sexual frustration
		Violence by drug dealers during the sale
		Institutional violence: involuntary confinement in rehabilitation centers, abuse of power, humiliation by health personnel, denial of medical care
		Stigma due to the use of crystal meth: being perceived as sources of STI/HIV infection and devalued as a person
**Perceived benefits**	Physical	More extraordinary physical and sexual performance: unlimited energy source
		Enhanced pleasure: greater motivation and sexual excitement
		Weight loss
	Mental	Increased ability to concentrate
		The feeling of euphoria, happiness
	Social	Relaxation during sex
		Sexual socialization
		Greater possibility to practice intense forms of sex without stigma or shame (e.g., bottoming, sadomasochism, fisting)
		Improved daily tasks and activities

### Narrated Experiences of Adverse Effects or Perceived Benefits of Substance Use

Experiences of gay male participants related to the perceived effects of substance use were connected to specific use environments (Table 5). For example, some respondents noted that the initiation or recreational drug environments were associated with experimental use and greater benefits, such as with marijuana. Notably, TP specifically highlighted the use of marijuana as beneficial for socialization, and JL noted its use to improve his mental health and overall well-being after experiencing an episode of extreme violence.

**Table 5:** Experiences of adverse effects and perceived benefits of substance use among gay men

	**Domain**	**Substance used**	**Quote**	**Substance use environment**
**Perceived benefits**	Physical	Poppers	*From what you said, if there are positive effects […] I started using poppers because they told me that it was easier for [guys to penetrate] me that way [laughs]. I enjoy being [a] bottom, but I struggle to do it. […] I had already done it [bottomed] before, so I know it is not [always] true, but inside, I feel that if I do not [use] poppers, I can no longer be [a] bottom* **(DP, 31 years old, northern region)**	Sexual drug environment
	Mental	Marijuana	*Just because of all the damage that the last year has left me, [it] was recommended [that I] smoke marijuana. That’s what I’ve been consuming right now when I have my anxiety attacks, my panic attacks, nights of terror and everything that has come from my traumas of the kidnapping. It’s what I end up smoking when I’m suddenly a little negligent and […] I kind of ground myself a little* **(JL, 32 years old, central region)**	Recreational drug environment
	Social	Marijuana	*That is where […] marijuana [use…] associated [with something] non-sexual comes from. My marijuana use is prolonged; I do not remember exactly how many years, but […] it did not affect me to the point that they […even] noticed in my house. At parties with family, when everyone was drinking or drunk, I just went out, [used marijuana], danced, [experiencing] euphoria [and] joy…* **(TP, 35 years old, central region)**	Initiation drug environment
**Adverse effects**	Physical	Crystal meth	*I think the main thing I noticed was the physical. […] I remember when I first started using meth, I weighed 58 kg, and when I decided to stop using meth, I weighed 52 kg. I lost 6 kg. [Also], I mean, all of a sudden, I literally saw in the toilet bowl that I was peeing almost like it was Coca Cola, and it smelled awful because I had used so much meth. I honestly don’t count how much meth I used, but [once] I used meth for three days straight* **(4AM, 31 years old, central region)**	Greater sexual pleasure drug environment
	Mental	Ethyl chloride	*It scared me a lot when my boyfriend started hallucinating. You are [being penetrated], and the guy is seeking people who are not there. First, it was an isolated episode, like [it lasted for] seconds […]. Later, I noticed a pattern: it [hallucinating] happens every time. It has scared me a lot because I do not know how […] these situations [might] escalate. Furthermore, it is also like, "Dude, I would rather not be around that," and we [ended dating after a few] months…* **(JQ, 34 years old, southern region)**	Sexual drug environment
	Social	Crystal meth	*… On the other hand, when I stop using, what happens to me a lot is that, at the end, I am super moody and annoyed. I fight with my friends, or I fight with my sexual partners because, after […] three days of not sleeping and using, it seems that my mental and emotional stability is bad. And then, something that happened to me frequently with a partner was that we would fight and fight after having had two super cool days. It was like a script: “Oh, you know that on Sunday night, or Monday morning, this is going to be shit”. I felt that it had to do with that use…* **(Car Ne, 31 years old, northern region)**	Greater sexual pleasure drug environment

What distinguishes substance use within sexual environments for participants in this study is that both the adverse effects and perceived benefits are directly associated with engagement in sexual activity. The effects of the substances reported in the *sexual drug environment*, or the *greater sexual pleasure drug environment* had greater importance for some gay men across their substance use experiences than those in the initiation or recreational drug environments. Perceived benefits were linked to sexual gratification and pleasure, increased sensitivity, and bodily satisfaction, mainly with marijuana and poppers. For example, the use of poppers for DP had physical benefits for penetrative ease during receptive anal sex (i.e., bottom) mainly due to dilation of the anal sphincter, resulting in greater pleasure sensation and sexual enjoyment. However, the most significant adverse effects perceived by gay men in the four substance use environments were related to substances used during sex or for greater sexual pleasure. In that sense, JQ described how excessive ethyl chloride use caused hallucinations and affected his relationship with his partner, just like Car Ne, for whom substance use from these environments (mainly crystal meth) influenced his mood and social relationships negatively*.*

One of the primary adverse effects of substance use reported by nearly all gay men was related to their mental health, mainly due to excessive use of crystal meth, which led to prolonged periods of sleep deprivation. Psychotic disorders, as well as auditory, visual, and sensory hallucinations, were also noted as consequences of excessive use of sexualized substances. Similarly, a considerable amount of the discourse on the effects of crystal meth focused on the physical implications of ongoing use, such as described by 4AM. Among these are the lack of appetite and weight loss, sleep disorders, muscle pain, constipation, tachycardia, injuries due to the type of use (e.g., inhaling), and more severe effects such as seizures due to polysubstance use with sildenafil, ethyl chloride, and alcohol.

Interestingly, participants did not express significant concerns about their sexual health in connection to using substances. Issues such as STIs and HIV seemed to be relevant to some participants only in the context of their HIV diagnosis or as part of a reflective exercise on sexual health prevention strategies. Consequently, perceived adverse effects or benefits regarding sexual health were seldom mentioned or viewed merely as a coping mechanism against the stigma or stress associated with an HIV diagnosis. Relatedly, 4AM reflected upon his experience:

*… At least my [mother] did not accept my HIV diagnosis. […] I began to consume crystal to be able to avoid the pain I felt from that rejection. […] I think that one of the great things that crystal consumption and the HIV diagnosis have left me is that it has allowed me to take my life to another place, which [may not have happened otherwise] [4AM is an activist in the field of HIV/STI prevention] (4AM, central region).*

The supplementary table (Appendix A) at the end of this study includes additional narratives related to the adverse effects and perceived benefits of substance use and their contextualization within specific substance use environments. The appendix provides a broader range of quotes detailing the specific experiences of younger and older gay male study participants.

## Discussion

In this study, the effects of substance use on the biopsychosocial well-being of gay men encompass two distinct perspectives that include perceived positive benefits and adverse effects. The first three substance use environments (*initiation drug environment*, *recreational drug environment*, *sexual drug environment*) were primarily associated with perceived benefits, particularly with substances such as marijuana, poppers, and recreational drugs. A few participants noted improvements in mental health and emotional well-being because of substance use. Additionally, substances were reported to increase sexual desire and more pleasurable receptive anal sex (e.g., bottoming). Furthermore, substance use was also perceived by participants as a way to enhance socialization in bars, clubs, and other social settings.

In contrast, excessive use of substances within sexual environments (e.g., ethyl chloride, poppers, and GHB/GBL) and for greater sexual pleasure (e.g., crystal meth) was associated with a range of adverse effects, including an impact upon physical (e.g., seizures, headaches, weight loss, insomnia, tachycardia, liver damage), mental health (e.g., hallucinations and psychotic episodes), and social domains (e.g., relationship difficulties, violence, and stigma).

Research examining the effect of substance use on gay men is often framed in terms of risks related to sexualized substance use. In this study, results showed that much of the harmful health effects were associated with the *sexual drug environment* and the *greater sexual pleasure drug environment environments* (especially the latter), with crystal meth use accounting for much of the adverse effects, which far outweigh the perceived benefits across physical, mental health, and social domains. Other studies on sexualized substance use have found some adverse effects on mental health and physical well-being, including injuries to the skin/veins due to intravenous use; cardiovascular complications; psychotic, depressive, and anxiety disorders; visual, kinesthetic, tactile, and auditory hallucinations; and suicide ideation and attempts (Batisse et al., 2022; Knoops et al., 2022; Moreno-Gámez et al., 2022). Likewise, while less discussed, the relationship between substance use and intimate partner violence among gay men has been reported (Duncan et al., 2018), as well as the adverse social effects of substance use, including stigma and rejection from peers and sexual partners (Davis-Ewart et al., 2023), which was also reflected upon as negative experiences associated with sexualized substance use in this study.

The results of this study align with existing research examining motivations and benefits of substance use among gay men related to pleasurable sexual experiences. For example, the perceived benefits of using marijuana and poppers aligns with the work of Parent et al. (2021) and Schwartz et al. (2020) as substances that improve pleasure, increase physical sensations, reduce inhibitions and anxiety, and allow for muscle relaxation during anal sex. The benefits reported in other research on crystal meth use relate to engaging in certain sexual practices without stigma and non-sexual benefits such as increased energy, the ability to focus, weight loss, and happiness (Davis-Ewart et al., 2023; Gish et al., 2020).

An unexpected result of this study was related to the impact of substance use on sexual health, which was not seen as harmful for gay male participants, even though the sexual context is often linked to use among this diverse population (Florêncio, 2021; Stanton et al., 2022). However, multiple studies describe the correlation between sexualized substance use and HIV (via condomless sex or sex with multiple partners) along with other STIs (Rodríguez-Bolaños et al., 2022; Takeuchi et al., 2023); it was not notable among study participants other than for situational reasons, including coping with a new HIV diagnosis, for specific sexual practices (e.g., anal sex), and related to self-care.

A possible explanation for this finding is that, socially, the sexual health care of gay men is often closely associated with HIV prevention (Koester et al., 2013). However, in our study, more than half of the participants were living with HIV, and half of those without HIV were taking PrEP, which minimized concerns about contracting HIV (Koester et al., 2017). Another potential explanation is that sexual *expression* may serve as a rebellious response to sexual *repression* linked to specific HIV prevention strategies (Íncera-Fernández et al., 2023). Ultimately, the prioritization of pleasure derived from the combination of substance use and sexual practices may have outweighed concerns about the risk of acquiring STIs (Gaspar et al., 2022).

Faced with a phenomenon as complex and multi-causal as the health-disease model when applied to a minority social group, and rather than delving into the moral binary of assessing “good” or “bad” health or risk behaviors, the focus should be on evaluating the usefulness of the findings for explanations or solutions to meeting the unique health, mental health, and sexual health needs of gay men in Mexico. Relatedly, Castiel and Álvarez-Dardet (2007) use the term “persecutory health” to refer to the secondary effect of health promotion that frames decisions related to one’s health solely dependent upon an individual without consideration for structural and systemic supports and connections or lack thereof.

In this regard, Gaspar et al. (2022) argue that it is essential to move beyond the risk-pleasure binary in studies related to substance use among gay men, drawing from the concept that perceived adverse effects or benefits may depend upon an individual’s specific relationship with substances. The risk-pleasure dichotomy may not manifest equally for each person. Therefore, it is necessary to elevate the voices of gay men and their uniquely lived experiences. This approach affords a more intimate analysis of perceived benefits or harms, including other emotional dimensions such as curiosity, expectations, fears, pain, and desires. In short, it highlights the role of gay men as agents in shaping their worldviews, their health-illness processes, connections to substance use, and the potential for resolution of their problems (Romaní, 2007).

This study lends supportive evidence for developing education and prevention strategies that address substance use among gay men that can positively impact their well-being, relationships, and sexual health. Study findings may also assist practitioners with developing affirming healthcare strategies that provide better support for their gay male clients navigating what they perceive to be the benefits and adverse effects of sexualized substance use in unique environments.

### Limitations

There are a number of limitations to the present study, including the inability to apply the results as generalizable to all gay men living in Mexico. The sample was also comprised of gay men who sought care in sexual healthcare centers for concerns related to HIV and substance use. However, the study results may reflect some gay male experiences from one culture (e.g., Mexican) and shared social dynamics (e.g., sexualized substance use). Another limitation pertains to the small sample size. Regardless, it remains essential that we understand several challenges faced by gay men living in Mexico, such as the stigma associated with substance use in combination with navigating pervasive forms of discrimination and homophobia. Moreover, such forms of oppression may have contributed to discouraging more gay men from talking openly about their substance use. Additionally, participants received no financial incentives for participating in this study, which may have dissuaded others from engagement.

### Public Policy Response

The National Commission Against Addictions (CONADIC) and the National Commission on Mental Health and Addictions (CONASAMA) are the two Mexican government institutions that create and implement public policies on prevention, education, treatment, and care for challenges related to tobacco, alcohol, and other substances based on standards that include universal access to health care, strengthening of community care, and protection of human rights (Secretaría de Gobernación, 1986, 2023). While these institutions offer primary care services, information portals, and referrals across the health sector, attention to the needs of sexual and gender-diverse populations (LGBTQ +) is scarce. The primary focus is also solely on the abstinence model, overlooking the great potential for harm reduction. Health and mental health service providers throughout Mexico, particularly CONADIC and CONASAMA, may find the harm reduction model beneficial, as it complies with the constitutional mandate of health protection and considers the unique health needs from the perspective of those utilizing substances or navigating addiction (Jargiełło, 2021).

## Conclusion

In conclusion, results from this study provide important insight into substance use experiences among Mexican gay men and the perceived benefits and harm to their health and well-being. A few perceived benefits related to the use of recreational substances were identified, such as an improvement in mental health and emotional well-being, heightened sexual experiences, and increased socialization. Oppositely, notable harmful effects were reported, particularly with crystal meth, related to a higher proportion of negative impacts upon physical, mental, and social health. This contrast highlights the complexity of decisions and experiences related to substance use among the gay male participants. In contrast, risks to health and interpersonal relationships were often conflicted with the pursuit of sexual pleasure and other perceived benefits.

Knowledge of the effects of substance use and sexual health among gay men may assist with health promotion efforts, harm-reduction strategies, public policies, and clinical support from the fields of medicine and psychology alike. Such interventions must also attend to the unique sociocultural aspects of practice with this diverse population and continually promote human rights, self-determination, non-discrimination, and health equity.

## Supplementary Information

Below is the link to the electronic supplementary material.

Supplementary file1 (DOCX 31 KB)

## Data Availability

Our research data used to support the findings of this study are not available because it is not part of the authorization granted by the Ethics Committee for this. Furthermore, it was not stipulated in the informed consent form that was presented to the participants.
