# Supplementary material for: Substance Use Among Gay Men Living in Mexico: Perceptions of Well-Being and Implications for Health
Source: Sex Res Social Policy. 2025 Mar 29;23(1):74–86. doi: 10.1007/s13178-025-01120-9 (PMC12971749; doi:10.1007/s13178-025-01120-9)
Supplement: Supplementary file 1 — Supplementary file1 (DOCX 31 KB) [file 13178_2025_1120_MOESM1_ESM.docx]

**Appendix A: Supplementary table. Experiences of Adverse Effects and Perceived Benefits of Substance Use Among Gay Men living in Mexico**

|  | Domain | Substance used | Quote | Substance use environment |
| --- | --- | --- | --- | --- |
| Perceived benefits | Physical | Poppers | *From what you said, if there are positive effects […] I started using poppers because they told me that it was easier for [guys to penetrate] me that way [laughs]. I enjoy being bottom, but I struggle to do it. […] I had already done it [bottomed] before, so I know it is not [always] true, but inside, I feel that if I do not [use] poppers, I can no longer be [a] bottom* (DP, 31 years old, northern region). | Sexual drug environment |
|  |  | Marijuana | *I used marijuana a lot for exercise. I feel better […] the movement of the muscle when I lift weights, or when I'm running. I last longer, or my performance improves when I use one or the other* (Neblino, 26 years old, northern region). | Recreational drug environment |
|  |  | Crystal meth | *It's like a shock of energy that runs through your body every time, but that effect diminishes, that's when people start to increase their consumption, but I would rather not get to that point […]. Basically, it's an unlimited source of energy…* (Metatrón, 40 years old, southern region). | Drug environment for greater sexual pleasure |
|  | Mental | Marijuana | *Just because of all the damage that the last year has left me, [it was] recommended to smoke marijuana. That’s what I’ve been consuming right now when I have my anxiety attacks, my panic attacks, nights of terror and everything that has come from my traumas of the kidnapping. It’s what I end up smoking when I’m suddenly a little negligent and […] I kind of ground myself a little* (JL, 32 years old, central region) | Recreational drug environment |
|  |  | Crystal meth | *… When we are […using] substances, we enter a [state] of euphoria* (AV, 36 years old, southern region). | Drug environment for greater sexual pleasure |
|  |  | Crystal meth | *… There was a time when I was trying to lose weight. I said, "Well, what will not eating do to me? Well, a smoker." Thus, […] to do some other activity that was not socializing, or was not sexualizing [laughs], that is how I came to use […] methamphetamine. In other words, smoking stops my hunger because before, I ate a lot out of anxiety. […] Strangely, the crystal does not give me that effect; it doesn't make me anxious; it calms me down and focuses me* (Neblino, 26 years old, northern region). | Drug environment for greater sexual pleasure |
|  |  | Marijuana | *I don't smoke, but I do use marijuana [in food] and I've done it for work. […] I felt like it was going to help me with certain things [and be] more calm... I did it a while ago, but I haven't done it again. At that time, I was really feeling very stressed, and I felt like it did help me* (DP, 31 years old, northern region). | Initiation drug environment |
|  | Social | Marijuana | *That is where […] marijuana [use…] associated [with something] non-sexual comes from. My marijuana use is prolonged; I do not remember exactly how many years, but […] it did not affect me to the point that they […even] noticed in my house. At parties with family, when everyone was drinking or drunk, I just went out, [used marijuana], danced, [experiencing] euphoria [and] joy...* (TP, 35 years old, central region). | Initiation drug environment |
|  |  | Tobacco | *…In high school, I was already smoking because I wanted to feel important, […] I wanted to stand out. Because yes, the truth is that I felt small, and they bullied me. Yes, they hurt me [and] hit me... I had to stand out […], and the way I [did this] was by smoking [tobacco]. I thought it made me seem important* (MC, 37 years old, central region). | Initiation drug environment |
|  |  | Alcohol, Tobacco, Marijuana, Mushrooms, LSD, Ecstasy | *…I think that alcohol, cigarettes, marijuana, or other drugs like mushrooms, LSD, or ecstasy I do not use […] in the context of sexuality; I use them more for parties and socializing. Yes, I drink a lot…* (Car Ne, 31 years old, northern region) | Recreational drug environment |
|  |  | Marijuana | *So, I never became a fan of substances until I started my sex life. It was with friends that I first tried marijuana, and then with a sexual partner. I did not know that he smoked marijuana, and then he offered it to me to have sex... and I said: "Well, okay." What happened? …the first experience was good! [Laughs] And, so I said, "Wow! I like it…this suits me"* (Neblino, 26 years old, northern region). | Sexual drug environment |
|  |  | Crystal meth | *…So, something similar happens with using crystal. […] [When using crystal], I can do certain practices [and explore them with greater interest]. […] Practices such as sadomasochism or fisting, [and using crystal makes me] more willing [to try new things]* (Car Ne, 31 years old, northern region). | Drug environment for greater sexual pleasure |
|  |  | Crystal meth | *I chose to face the fact that my family, or at least my [mother], did not accept my HIV diagnosis. […] I began to consume crystal to be able to avoid the pain I felt from that rejection. […] I think that one of the great things that crystal consumption and the HIV diagnosis have left me is that it has allowed me to take my life to another place, which [may not have happened otherwise] [4AM is an activist in the field of HIV/STI prevention]* (4AM, 31 years old, central region). | Drug environment for greater sexual pleasure |
|  |  | Ecstasy | *For example, in the case of tachas [ecstasy], they made me social, they put me in a happy, joyful mood, “I am everyone's friend” and I invite you to my house, we're going to have a party, and we'll see what day we're going to eat...* (TP, 35 years old, central region). | Recreational drug environment |
| Adverse effects | Physical | Poppers | *There came a point where I […] [used too] many poppers […and] the headaches that come are horrible […]. There was once when I had to stay locked up in my room all day with the lights off in bed because I was very sick. They call it "your brain goes cold due to poppers"* (SM, 22 years old, northern region). | Sexual drug environment |
|  |  | Crystal meth | *I think the main thing I noticed was the physical. […] I remember when I started using meth, I weighed 58 kg, and when I decided to stop using meth, I weighed 52 kg. I lost 6kg. [Also], I mean, suddenly, I literally saw in the toilet bowl that I was peeing almost like it was Coca-Cola, and it smelled awful because I had used so much meth. I honestly don’t count how much meth I used, but I used meth for three days straight* (4AM, 31 years old, central region). | Drug environment for greater sexual pleasure |
|  |  | Crystal meth | *… when I began to consume ice [crystal] a lot, I noticed many things. The first is that you hardly eat. Your hunger [and] sleep are inhibited. [At times] you start with a “fucking psychosis”, […] tremors, the rattling of bones, your muscles start to hurt, [...] you get constipated.* (JL, 32 years old, central region). | Drug environment for greater sexual pleasure |
|  |  | Crystal meth | *… After [I] smoked crystal [I moved to] injected crystal, the slam, […] is when my life completely took a nosedive. I lost jobs and started to steal things from the house to feed this vice. Practically, all of 2020 [...] I was in [a rehabilitation center] because there was no way [for] me to stop. […] I did not recognize when [...] people told me, “You are skinny; what is wrong with you?” I even got upset; I said, “What the hell do they care?” [...]. It is fascinating how sometimes you cannot realize how people see you because when I showered one day, I looked in the mirror and said: “It is no wonder people are [surprised] when they see me; I am [all] bones” […]. I heard the concern of my relatives and did nothing but take drugs* (TP, 35 years old, central region). | Drug environment for greater sexual pleasure |
|  |  | Crystal meth | *I had a meth psychosis where I swore my heart was going to explode. I got to a heart rate of 155, and I did some research at the time, and it turned out I was already at risk for a heart attack, at 160 beats per minute. So, well, my anxiety went through the roof…* (SM, 22 years old, northern region). | Drug environment for greater sexual pleasure |
|  |  | Crystal meth | *… I started to have liver problems, […] I had grade one hepatic steatosis […], so he sent me to the gastro [Gastroenterologist]. My infectious disease specialist thought: "maybe this boy has Hepatitis B or C." The gastro tested me for Hepatitis B and C. I tested positive for Hepatitis C even though I have never had a slam [injecting meth]. When he starts to ask me about my sexual practices, substance use […] I tell him: "I have used crystal meth for so long, in these modalities…"* (4AM, 31 years old, central region). | Drug environment for greater sexual pleasure |
|  |  | Crystal meth | *I’m not going to demonize drugs, I am not going to put myself in a puritanical position and say: “It is wrong, don’t do it.” I just […], from my perspective, I would not like to go back to that because I already know that my life is affected in a very [negative] way: at work, in my family and in my physique, therefore, in my health.* (TP, 35 years old, central region). | Drug environment for greater sexual pleasure |
|  | Mental | Crystal meth | *I had a meth psychosis where I swore my heart was going to explode. I got to a heart rate of 155, and I did some research at the time, and it turned out I was already at risk for a heart attack, at 160 beats per minute. So, well, my anxiety went through the roof…* (SM, 22 years old, northern region). | Drug environment for greater sexual pleasure |
|  |  | Ethyl chloride | *It scared me a lot when my boyfriend started hallucinating. You are [being penetrated], and the guy is seeing people who are not there. First, it was an isolated episode, like [it lasted for] seconds […]. Later, I noticed a pattern: it [hallucinating] happens every time. It has scared me a lot because I do not know how […] these situations [might] escalate. Furthermore, it is also like, “Dude, I would rather not be around that”, and we [ended dating after a few] months...* (JQ, 34 years old, southern region). | Sexual drug environment |
|  |  | Crystal Meth | *… when I began to consume ice [crystal] a lot, I noticed many things. The first is that you hardly eat. Your hunger [and] sleep are inhibited. [At times] you start with a “fucking psychosis”, […] tremors, the rattling of bones, your muscles start to hurt, [...] you get constipated.* (JL, central region). | Drug environment for greater sexual pleasure |
|  |  | Crystal Meth | *However, that same euphoria comes with […] dopamine suppression, so we enter [depression or anger] […]. Just as pleasure is also increased, emotions are increased. So, complicated situations [arise], such as the distortion of reality […]. The senses of sight, smell, and touch are distorted; we [often experience a] different reality. […] [This can result in] paranoid situations. […] When an idea enters your mind, it is not necessarily real, but it could be.* (AV, 36 years old, southern region). | Drug environment for greater sexual pleasure |
|  |  | Crystal meth | *When I came back to Mexico, I managed to have a forced abstinence, […] because I was not conscious. […] I don’t know how it [happened], but then it started to be [necessary] to relate to others. Any kind of relationship requires consuming [meth] to even talk to another man. I need to consume because I don’t feel enough, capable or safe, that is, it starts to generate more insecurities than personal strengths that I may or may not have had…* (RO, 32 years old, central region). | Drug environment for greater sexual pleasure |
|  |  | Crystal meth | *Being "encristalado" (under the influence of the crystal) in places you don't know, with people you don't know, the effect of the crystal on me was paranoia...* (MC, 37 years old, central region). | Drug environment for greater sexual pleasure |
|  |  | Crystal meth | *I once saw a guy in a motel who started telling me that he had brought 20 people […]. He had been awake for days, and he was also shooting up […]. I realized that he was seeing that there were 20 guys in the motel room, and he said: “Look, they are climbing down the windows! What is the motel owner going to say? They are going to take us to the police!” I was actually scared* (TP, 35 years old, central region). | Drug environment for greater sexual pleasure |
|  | Social | Crystal meth | *… On the other hand, when I stop using, what happens to me a lot is that, at the end, I am super moody and annoyed. I fight with my friends, or I fight with my sexual partners because, after […] three days of not sleeping and using, it seems that my mental and emotional stability is bad. And then, something that happened to me frequently with a partner was that we would fight and fight after having had two super cool days. It was like a script: “Oh, you know that on Sunday night, or Monday morning, this is going to be shit”. I felt that it had to do with that use…* (Car Ne, 31 years old, northern region). | Drug environment for greater sexual pleasure |
|  |  | Alcohol | *When I lived in Mexico City, between the ages of 19 and 22, I did consume much alcohol, not to the point of perhaps consuming it every day, but every weekend and much more than I consume now at an ordinary party. Even so, there are times when I do consume a lot, but at that time, it was significantly related to alcohol and sex, [...] although it was basically with my partner at the time [...]. Moreover, it was that we both got “hasta la madre” [excessive consumption] […] and it was not good sex […] with alcohol. It must be a certain level [not excessive use], [because] if it is too much, [sex does not happen] anymore... (DP, northern region).* | Sexual drug environment |
|  |  | Crystal meth | *… After the smoked crystal [moved to] injected crystal, the slam, […] is when my life completely took a nosedive. I lost jobs and started to steal things from the house to feed this vice. Practically, all of 2020 [...] I was in [a rehabilitation center] because there was no way [for] me to stop. […] I did not recognize when [...] people told me, "You are skinny; what is wrong with you?" I even got upset; I said, "What the hell do they care?" [...]. It is fascinating how sometimes you cannot realize how people see you because when I showered one day, I looked in the mirror and said: "It is no wonder people are [surprised] when they see me; I am [all] bones" […]. I heard the concern of my relatives and did nothing but take drugs* (TP, 35 years old, central region). | Drug environment for greater sexual pleasure |
|  |  | Crystal meth | *After the kidnapping, because of the trauma it caused me, well, you know how it is “you put a pig near the mud” and I just went off again like “gorda en tobogán” [He just let himself go with crystal meth use]. I relapsed. Honestly, I could say I barely remember anything from last year, like, everything just went to shit terrible. It wasn't until late October-November that I started to ask myself: 'Dude, what the hell? What the hell is going on with you?* (JL, 32 years old, central region). | Drug environment for greater sexual pleasure |
|  |  | Crystal meth | *When I came back to Mexico, I managed to have a forced abstinence, […] because I was not conscious. […] I don’t know how it worked here, but then it started to be [necessary] to relate to others. Any kind of relationship required consuming to even talk to another man. I need to consume because I don’t feel enough, capable or safe, that is, it starts to generate more insecurities than personal strengths that I may or may not have had…* (RO, 32 years old, central region). | Drug environment for greater sexual pleasure |
|  |  | Crystal meth | *So, that shit lasted a long time… […] I’m almost 2 years in, but it was exponential. The truth is that it broke me […]. Because of course, my partner kicked me out of the house, my family now distrusts what I do, what I say, they don’t know if I’m using or not. I mean, the truth is that it killed many things in many areas of life.* (MC, 37 years old, central region). | Drug environment for greater sexual pleasure |
|  |  | Crystal meth | *More in the context of here in Nuevo Leon, […] there are many situations where […] they steal from them and […] things are taken from them. […] Other contexts that have nothing to do with what one expects…* (Neblino, 26 years old, northern region). | Drug environment for greater sexual pleasure |
|  |  | Crystal meth/  Polysubstance use | *Back in Mexico City, I got back together with my partner. We lived together, and the truth is that at that time I went like “a thread of stocking”, [that is], I went from one substance to another [...]. From there my relationship started to have problems related to my substance use and also with his substance use because my partner also started using substances, but each one did it on their own. We had numerous issues with the relationship. Furthermore, within the relationship, there were like 3 other people, that is, there were 5 of us in a polyamorous relationship. The truth is that it was a bit horrible…* (AV, 36 years old, southern region). | Drug environment for greater sexual pleasure |
